# Modeling truncated pixel values of faint reflections in MicroED images^1^

**DOI:** 10.1107/S1600576716007196

**Published:** 2016-05-11

**Authors:** Johan Hattne, Dan Shi, M. Jason de la Cruz, Francis E. Reyes, Tamir Gonen

**Affiliations:** a Janelia Research Campus, Howard Hughes Medical Institute, Ashburn, VA 20147, USA

**Keywords:** cryoEM, micro-electron diffraction, MicroED, X-ray free-electron lasers, XFELs

## Abstract

The weak pixel counts surrounding the Bragg spots in a diffraction image are important for establishing a model of the background underneath the peak and estimating the reliability of the integrated intensities. Under certain circumstances, particularly with equipment not optimized for low-intensity measurements, these pixel values may be corrupted by corrections applied to the raw image. This can lead to truncation of low pixel counts, resulting in anomalies in the integrated Bragg intensities, such as systematically higher signal-to-noise ratios. A correction for this effect can be approximated by a three-parameter lognormal distribution fitted to the weakly positive-valued pixels at similar scattering angles. The procedure is validated by the improved refinement of an atomic model against structure factor amplitudes derived from corrected micro-electron diffraction (MicroED) images.

## Abstract 2

A procedure is presented to model the truncated low pixel counts in micro-electron diffraction (MicroED) images. The correction could extend to any conventional macromolecular X-ray crystallography or X-ray free-electron laser measurements.

## 1. Introduction   

The success of diffraction data analysis, and consequently the quality of the final atomic model, hinges on accurate integration of the recorded Bragg reflections. The intensities of these reflections decrease with increasing scattering angle until the point where their peaks become indistinguishable from the surrounding background (Bourenkov & Popov, 2006 ▸). Ignoring the effects of solvent scattering and artifacts such as ice rings (Glover *et al.*, 1991 ▸), the recorded counts of pixels between the Bragg spots follow the same general pattern; the greater the distance from the intersection point of the direct beam with the detector surface, the smaller their values. Because the background pixels around a reflection are commonly used to estimate the noise contribution to the integrated signal (Leslie, 1999 ▸), successful data reduction generally requires that all pixel values are accurately recorded, irrespective of their scattering angle and magnitude, or whether they represent Bragg spots or not.

Many detector systems used to record diffraction data apply corrections to the raw data before a rectified image is presented to the experimenter for processing. The flat-field calibration is one such correction. For CCD- and CMOS-based detectors, this two-step procedure consists of dark-frame correction, where a previously recorded, unexposed image is subtracted, followed by multiplication with a gain image. Dark-frame correction removes features that arise from the small currents that flow through the sensor even when the shutter is closed. The subsequent gain correction compensates for the uneven response of individual pixels by ensuring that the calibrated readout under uniform flat-field illumination is featureless. In some cases, images are uninterpretable unless these corrections are applied.

**Figure 1:**
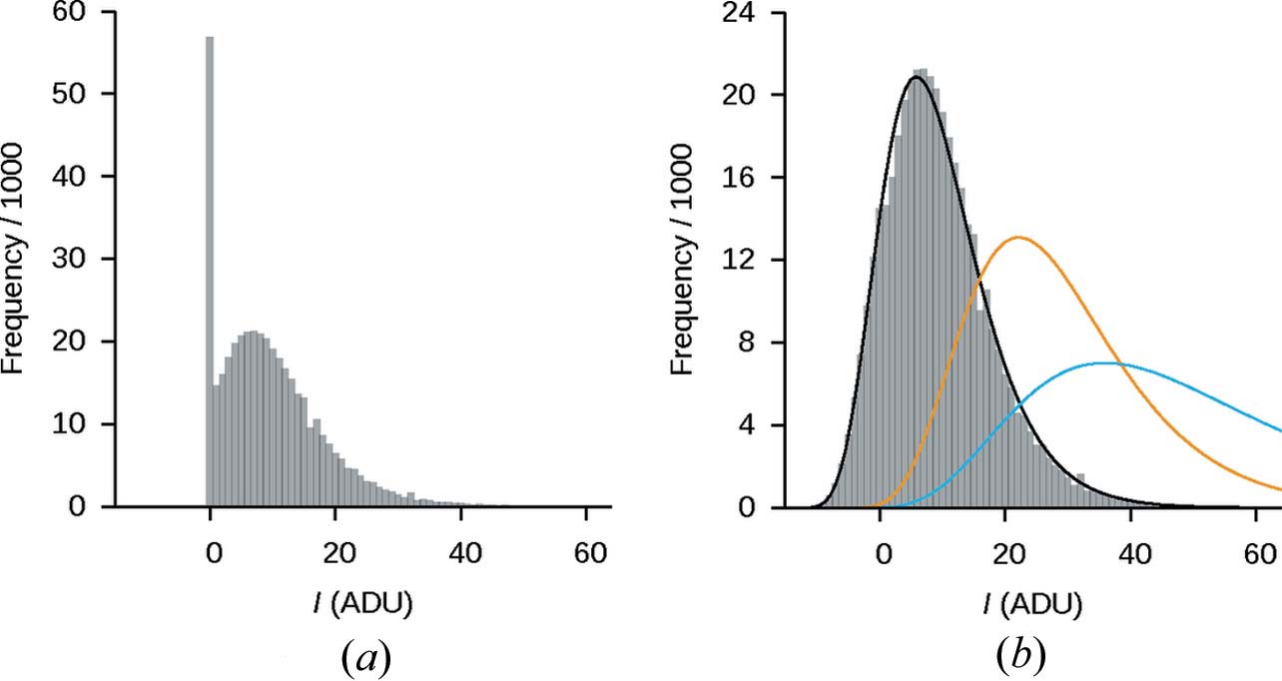
Distribution of the low counts in a typical MicroED image of proteinase K collected by continuous rotation using the rolling shutter mode of the camera. (*a*) The histogram in the second outermost shell between 1.5 and 1.7 Å for an uncorrected image, and (*b*) the histogram of the corresponding corrected image. The continuous curves in (*b*) show the fitted lognormal distributions in the two innermost (resolutions lower than 4.7 Å in blue, resolutions between 3.4 and 4.7 Å in orange) and the second outermost (black curve) resolution shells. As the resolution increases, the mode and the variance of the distribution decrease.

A number of macromolecular crystal structures have recently been solved by micro-electron diffraction (MicroED) (Shi *et al.*, 2013 ▸; Nannenga, Shi, Hattne *et al.*, 2014 ▸; Rodriguez *et al.*, 2015 ▸; Yonekura *et al.*, 2015 ▸). In our laboratory, diffraction datasets have been recorded by continuous rotation (Nannenga, Shi, Leslie & Gonen, 2014 ▸) using a TVIPS TemCam-F416 CMOS camera. During data collection the crystal is slowly rotated in the electron beam and the accumulated counts are rapidly read out at regular intervals without interrupting the rotation of the sample. However, the camera’s ‘rolling shutter’ mode (Stumpf *et al.*, 2010 ▸) that makes these measurements possible is primarily intended to provide real-time visual feedback during data collection. The camera does apply a flat-field correction, but the storage format required to sustain the high data-transfer rates is restricted to representing pixel values as unsigned 16 bit integers. This causes problems for weak reflections, which are typically observed at high resolution. Around these reflections the raw counts on the detector may be comparable in magnitude to those in the dark frame. Owing to random fluctuations in the raw counts, dark-frame subtraction may then yield very small or even negative values, which are propagated through the subsequent gain correction. As negative counts cannot be represented in the storage format, they are truncated to zero, and information about the true, negative value is lost. Generally, the effect is not immediately apparent on visual inspection of the diffraction pattern, but becomes clear in histograms of the low pixel values, which feature a prominent peak at zero analog-to-digital units (ADU) (Fig. 1 ▸ *a*).

It is conceivable that the dark frame could be offset by some constant to reduce the probability that dark subtraction yields a negative number. This is not easily achievable without altering the software used to control the camera. Modifying the camera’s storage format to use signed integers is similarly impractical. Disabling the flatfield correction altogether is unattractive, since it would remove the ability to view calibrated diffraction images while they are being retrieved from the camera. The remaining option is to attempt to recover as much information as possible from the dataset. Here we present a procedure to model the values of the truncated pixels with zero counts from the histogram of the values of the remaining pixels.

## 2. Methods   

For a sufficiently large sample of weakly positive-valued pixels, their histogram allows the distribution of the counts around zero to be modeled. For diffraction patterns, the parameters of the distribution of recorded counts across the image depend on the scattering angle (Fig. 1 ▸ *b*). Therefore separate models are derived from pixels within a narrow interval of scattering angles. The finite range of scattering angles leads to heavy-tailed distributions, particularly at low resolution where a larger spread of scattering angles is necessary to provide an adequate sample size to model the distribution. Invalid pixels, for example pixels in the shadow of the beam stop, are not considered because they do not follow the distribution of pixels that record electrons scattered from the sample.

We use the lognormal distribution to model the behavior of the low-valued pixels. The lognormal distribution is expected where the observed counts are the result of independent multiplicative processes in the detector (Kissick *et al.*, 2010 ▸), but in our case its use is primarily motivated by its quality of fit to the experimental data (mean r.m.s.d. 327 ADU). The probability density function *f* and cumulative distribution function *F* of the lognormal distribution are given by 
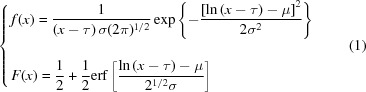
where μ and σ are the location and scale parameters, respectively. A third parameter, τ, is used to arbitrarily shift the distribution, which allows the random variable it models to take any real value 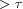, rather than just positive values. Assuming the pixels in a given resolution range of a diffraction image are independent and identically distributed, the probability of observing a pixel with true integer count *I*, such that 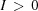, can then be approximated:
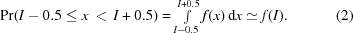
The probability of observing a pixel with any value 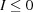 is given by 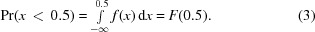
Let 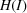 denote the number of pixels with value *I* in the image. For any integer count *I* in the closed interval 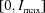, 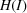 defines the observed histogram (Fig. 1 ▸). We assume that any pixel with 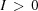 is measured correctly; a pixel with 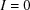 could represent either a true value of zero or a negative value. We seek the parameters μ, σ and τ that maximize the probability of observing 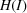. This is equivalent to maximizing the likelihood, or more conveniently, the log-likelihood, which in our model is given by 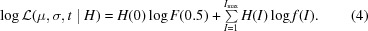
This can be done using standard optimization algorithms such as the BFGS implementation in the R environment (R Core Team, 2015 ▸).

**Figure 2:**
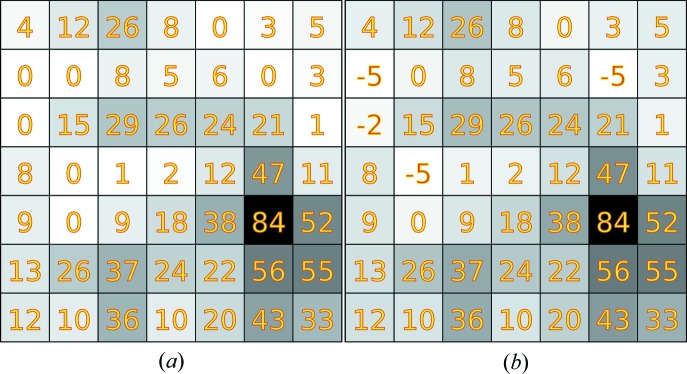
A spot near the edge of the detector (*d* = 1.8 Å) (*a*) before and (*b*) after correction. Pixels with initial counts >0 ADU are otherwise unchanged, while zero-valued pixels exhibit counts ≤0 ADU in the corrected image.

The recovered parameters define the maximum likelihood lognormal distribution corresponding to the observed histogram in the given resolution shell. Negative values are then randomly assigned to the 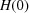 pixels that were initially zero, such that the histogram for 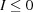 in the corrected image conforms to the fitted distribution (Fig. 1 ▸ *b*). Pixels with initially positive values remain unchanged (Fig. 2 ▸), and the frequency of negative values agrees with the optimized model. Only the spatial arrangement of the negative values is random. Uncorrected images that do not contain any zero-valued pixels will have 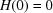; the correction does not alter these images in any way.

If the corrected image will be stored in a format that does not support negative counts (*e.g.* SMV), an offset has to be applied before the image is output. To preserve correct integration downstream, the integration software has to be made aware of this offset (*e.g. ADCOFFSET* in *MOSFLM*). Choosing the offset as the negated value of the smallest count in all resolution shells of all images after correction allows straightforward processing of the sweep.

The procedure was validated against MicroED images collected from four crystals of proteinase K. Protein solutions from *Engyodontium album* (Sigma-Aldrich, St Louis, MO, USA) were prepared by combining 2 µl of protein solution (50 mg ml^−1^) with 2 µl of precipitant solution (1.0–1.3 *M* ammonium sulfate, 0.1 *M* Tris pH 8.0). Crystals in space group *P*4_3_2_1_2 with unit cell *a* = *b* = 67.3, *c* = 101 Å appeared in hanging drops after equilibrating against the precipitant solution for three days. MicroED images were recorded on a transmission electron microscope (FEI) equipped with a field emission gun and a TVIPS TemCam-F416 CMOS camera using published protocols (Nannenga, Shi, Leslie & Gonen, 2014 ▸; Shi *et al.*, 2016 ▸). At an acceleration voltage of 200 kV and a camera length of 1.2 m (corresponding to a virtual detector distance of 2.2 m) the detector can record reflections at resolutions up to ∼1.75 Å at the edges and ∼1.25 Å in the corners. The correction was applied to the images independently in ten concentric annuli of approximately equal area.

Corrected datasets were indexed and integrated with *MOSFLM* (Leslie & Powell, 2007 ▸). To ensure comparable integration for the uncorrected and corrected datasets only the missetting angles were optimized during integration. The mosaicity was refined to convergence for each crystal separately and then held constant during integration. All detector parameters were fixed, and the measurement box was set to a 13 × 13 pixel box with a 4 pixel border and an 8 pixel corner cutoff (Leslie, 1999 ▸). To allow the integration box to contain zero-valued pixels for the uncorrected data, *MOSFLM*’s NULLPIX parameter was set to −1. The intensities calculated by summation integration were scaled and merged using *AIMLESS* with default parameters (Evans & Murshudov, 2013 ▸). The upper resolution limit imposed during scaling lies just inside the detector corners where the number of observations is barely large enough to permit merging statistics to be calculated. This is beyond commonly employed resolution cutoffs, but allows the effect of the correction on the weakest high-resolution reflections to be evaluated.

**Figure 3:**
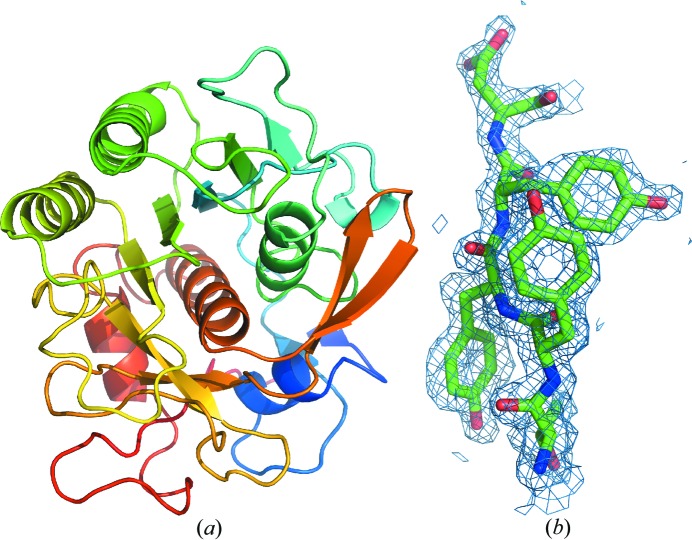
MicroED structure of proteinase K at 1.75 Å resolution. (*a*) The overall MicroED structure of proteinase K. (*b*) A five-residue fragment of the final model refined against the data derived from the corrected images. The SA composite omit map at 1.75 Å resolution is contoured at 1.0σ above the mean and shows a hole in the center of the tyrosine side chain. The figures were generated using *PyMol* (Schrödinger, 2014 ▸).

The merged data were phased by molecular replacement in *MOLREP* (Vagin & Teplyakov, 1997 ▸) using PDB ID 4woc (Guo *et al.*, 2015 ▸) as a search model, resulting in contrast scores of 27.57 and 32.56 for the uncorrected and corrected data, respectively. Both models were refined with *phenix.refine* (Afonine *et al.*, 2012 ▸) using electron scattering factors (Colliex *et al.*, 2006 ▸), automatic water modeling and weight optimization of the stereochemistry terms. Only reflections up to 1.75 Å were included in the refinement, because the completeness of the merged dataset drops rapidly beyond the edges of the detector [see Fig. 5(*a*) in §3]. The simulated annealing (SA) composite omit map computed by *CNS* (Brunger, 2007 ▸) clearly reveals depressions or even holes in the centers of the aromatic side chains (Fig. 3 ▸).

## 3. Results and discussion   

**Figure 4:**
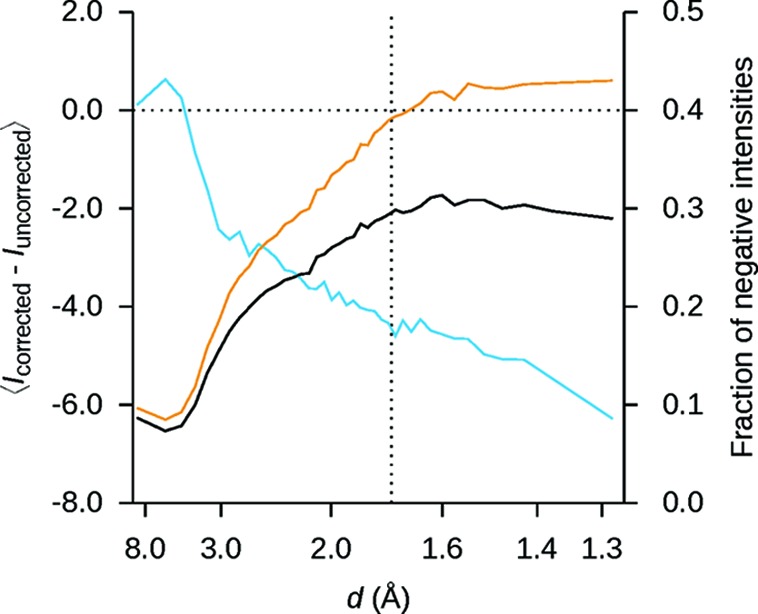
Effect of the procedure on the integrated intensities before scaling and merging. The average change in the integrated unmerged intensities (blue curve) is smoothly varying as a function of resolution. Except for at the lowest resolutions, the intensities are consistently lower in the corrected data, and the magnitude of the difference increases with resolution. The horizontal dotted line at 〈*I*_corrected_ − *I*_uncorrected_〉 = 1 is added to aid comparison. The fraction of negative intensities is larger in the corrected data (orange curve) than in the uncorrected data (black curve). The difference increases steadily until just beyond the edge of the detector, which is marked by a vertical dotted line.

The correction only modifies the zero-valued pixels in an image and it can never increase their values. Because the mode of the fitted distribution tends to decrease with increasing resolution (Fig. 1 ▸ *b*), the number and magnitude of the negative-valued pixels is expected to increase toward the edges of the detector. This behavior is seen in the integrated reflections (Fig. 4 ▸), with the exception of the low-resolution reflections, where the decreased values of the pixels surrounding the peaks lead to stronger integrated intensities after background subtraction. For higher-resolution reflections, where corrected pixels may fall within the foreground, the integrated intensities decrease as well. The magnitude of the difference between the integrated intensities before and after correction increases with resolution, and the corresponding increase in the fraction of negative intensities (Fig. 4 ▸) is consistent with this observation.

**Figure 5:**
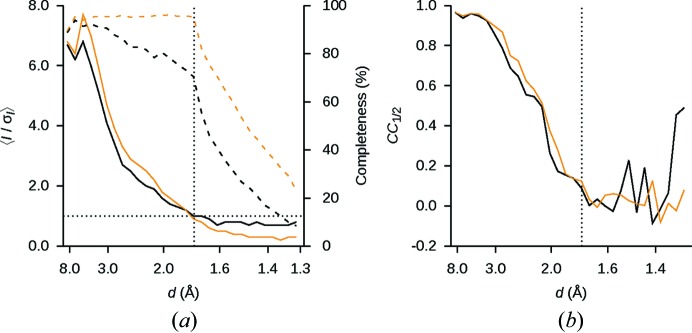
Merging statistics as a function of resolution. (*a*) At high resolution 〈*I*/σ*_I_*〉 is higher in the uncorrected dataset (black curve) than in the corrected dataset (orange curve), and the values tend to zero only in the corrected dataset. The horizontal dotted line at 〈*I*/σ*_I_*〉 = 1 is added to aid comparison. Beyond the edge of the detector (vertical dotted line) the completeness drops sharply for both the uncorrected (black dashed curve) and the corrected (orange dashed curve) datasets. (*b*) CC_1/2_ is slightly higher for the corrected images (orange curve) than for the uncorrected images (black curve). Beyond the edge of the detector, indicated by the vertical dotted line, the curves are dominated by noise.

Compared to the uncorrected images, the corrected dataset merged ∼2.5× more reflections (Table 1 ▸). The vast majority of the rejections for the uncorrected images occur during integration owing to excessive background gradient (87%), indicating problems modeling the background, where low pixel counts are more abundant. Other rejections are mostly due to incompletely recorded, partial reflections and ill-fitting peaks. The smaller number of outlier rejections in the corrected dataset is reflected in an increased completeness and multiplicity (Fig. 5 ▸ *a* and Table 1 ▸).

Except for the reflections only observed in the corners of the detector, the half-set correlation, CC_1/2_ (Karplus & Diederichs, 2012 ▸), is marginally higher for the corrected images than for the uncorrected images (Fig. 5 ▸ *b*). Beyond the edge of the detector CC_1/2_ is dominated by noise. The merging *R* factors on the other hand are higher for the corrected dataset than for the uncorrected images, and this is most pronounced in the higher-resolution shells. At high resolution, individual pixel counts are more affected by noise, and their variance is governed by fluctuations around low counts. In the uncorrected dataset these fluctuations are diminished when negative pixel counts are truncated, leading to artificially homogenous integrated intensities and underestimated standard deviations for the very weakest Bragg spots. The correction recovers some of this variance, and notably, 〈*I*/σ*_I_*〉 in the highest-resolution shell, where reflections are not visually discernible, drops twofold (Fig. 5 ▸ *a* and Table 1 ▸).

**Figure 6:**
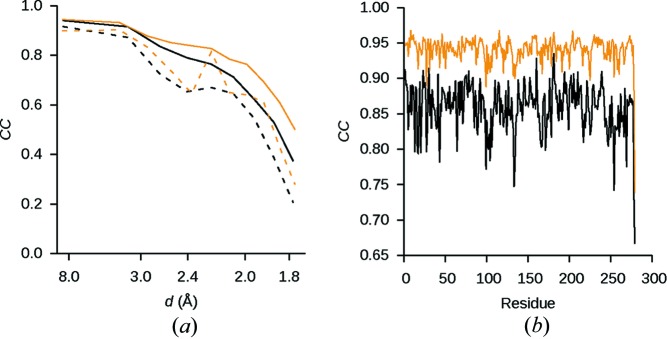
Correlation coefficients of the refined model. (*a*) Particularly at high resolution, CC_work_ (solid curves) and CC_free_ (dashed curves) are generally higher for the model refinement against the corrected dataset (orange curves) than for the model refined against the uncorrected dataset (black curves). (*b*) For all 279 residues of proteinase K, the real-space correlation coefficient for the corrected data in the resolution range between 1.75 and 5.00 Å is higher for the model refined against the corrected data than for the model refined against the same resolution range of the uncorrected data.

With otherwise identical protocols, the overall *R*_work_ and *R*_free_ values are lower by 1.0 and 0.7%, respectively, for the model refined against the corrected dataset compared to those for the uncorrected dataset. The correlation coefficients between the observed and calculated structure factor amplitudes are generally higher for the model refined against the corrected data than for the model refined against the uncorrected data, and the effect is more pronounced at higher resolution (Fig. 6 ▸ *a*). Similarly, the atomic model refined against the corrected data correlates better to its density map calculated from reflections in the interval between 1.75 and 5.00 Å than the model refined against the corresponding uncorrected data (Fig. 6 ▸ *b*). However, the atomic coordinates of the two models are very similar with an r.m.s.d. of 0.080 Å.

## 4. Conclusion   

The systematic truncation of weak pixel values introduces subtle anomalies in the integrated Bragg intensities, which propagate to the refined model. In the present case, the artifacts are due to the data format’s inability to represent negative counts. File formats restricted to unsigned integers are common in crystallography, but it is conceivable that similar problems could arise by other means. However, modeling the counts of the low-valued pixels can help to recover the true signal for the high-resolution reflections. For stronger reflections, the benefit of the correction lies mainly in a realistic appearance of the background surrounding the peak, which provides a more accurate estimate of its reliability. The end effect is that the merged reflections better represent the amplitudes of the diffracting crystal’s scattering factors. This in turn improves the quality of the final atomic model. Depending on the particular implementation of the spot-finding routine, the correction can also boost autoindexing and unit-cell determination of faint diffraction datasets, where an artificially flat background otherwise yields many spurious spots.

It must be noted that the pixel values that are lost in truncation can never be truthfully recovered. Future advances could improve the quality of the procedure introduced here, but the correct negative values of the affected pixels are fundamentally irretrievable. The procedure instead models the corrupted counts, which limits the accuracy of the correction to the quality of the model and the process used to determine its parameters. While the reliance on a random number generator for the spatial distribution of negative counts is appropriate since it models the stochastic fluctuations that initially lead to the negative, truncated pixel values, it implies that the procedure is non-deterministic. Owing to the local homogeneity of the detector, initial attempts at exploiting per-pixel statistics instead for the assignment of the negative counts have not been successful. However, separately applying the correction to smaller regions can reduce the impact of the random number generator. The current implementation limits the structure of these areas to concentric annuli, but this could be extended to arbitrary shapes, which together cover the surface of the detector.

Ideally, a diffraction measurement would be conducted such that the need for the correction described here would never arise. In emerging methods such as MicroED, which often rely on hard- and software originally developed and optimized for different purposes, this is not always immediately possible. Future developments in MicroED will address these difficulties by, for example, determining how to use the camera in a different mode that allows signed integers to be recorded.

The corrected data and the model refined against them are available under PDB id 5i9s and EMDB id EMD-8077. The uncorrected data have been deposited with the Structural Biology Data Grid (Meyer *et al.*, 2016 ▸) under doi 10.15785/SBGRID/262. The procedure will be included in an upcoming release of our conversion tools for MicroED diffraction images (Hattne *et al.*, 2015 ▸).

## Figures and Tables

**Table 1:** Merging and refinement statistics for the uncorrected and corrected datasets of proteinase K Both datasets were derived from the same 184 images collected from four separate nanocrystals of proteinase K. The frames were exposed for ∼4 s while the stage on which the crystals were mounted was continuously rotated at 0.09° s^−1^. The models derived from the uncorrected and corrected images contain 166 and 133 water molecules, respectively. Both models include two sulfate ions. For CC_1/2_ > 0.30, *AIMLESS* estimates the resolution limits to be 2.01 and 1.96 Å for the uncorrected and corrected datasets, respectively. The corresponding limits for 〈*I*/σ*_I_*〉 > 1.50 are 1.96 and 1.91 Å. Numbers in parentheses refer to the highest-resolution shell for either merging or refinement.

	Uncorrected	Corrected
Merging to 1.30 Å		
Resolution (Å)	21.91–1.30 (1.32–1.30)	21.91–1.30 (1.32–1.30)
*R*_merge_	0.329 (0.513)	0.629 (2.671)
*R*_r.i.m._	0.403 (0.726)	0.727 (3.627)
*R*_p.i.m._	0.225 (0.513)	0.347 (2.429)
CC_1/2_	0.896 (0.490)	0.842 (0.080)
Total No. of observations	61 731 (254)	154 259 (1083)
〈*I*/σ*_I_*〉	2.2 (0.8)	2.0 (0.3)
Wilson *B* (Å^2^)	16.4	17.2

Refinement to 1.75 Å		
Resolution (Å)	19.62–1.75 (1.82–1.75)	20.51–1.75 (1.81–1.75)
Completeness (%)	80.8 (71.2)	94.1 (94.0)
Multiplicity	2.5 (1.9)	4.5 (4.3)
*R*_work_ (%)	22.7 (36.5)	21.7 (34.8)
*R*_free_ (%)	27.3 (44.2)	26.6 (41.8)
